# It’s Yet Another Thing: Barriers to and Recommendations for Physician Referrals to Home-Based Palliative Care

**DOI:** 10.1093/geroni/igab046.635

**Published:** 2021-12-17

**Authors:** Alexis Coulourides Kogan, Valeria Cardenas, YuJun Zhu, Jenna Giulioni, Anna Rahman, Susan Enguidanos

**Affiliations:** 1 USC Keck School Of Medicine, Alhambra, California, United States; 2 University of Southern California, SOUTH PASADENA, California, United States; 3 USC Division of Biokinesiology and Physical Therapy, Los Angeles, California, United States; 4 University of Southern California, Los Angeles, California, United States

## Abstract

To understand primary care providers’ (PCPs) experiences with referring patients to home-based palliative care (HBPC), we conducted individual, key-informant interviews with 31 PCPs. About half participants were male (54.8%), White (42.5%), US-born (58.1%), and were 57 years old (SD=9.17), on average. About one-third of participants (32.3%) indicated they refer 10+ patients annually to HBPC, while most (80.7%) reported “strong” comfort discussing palliative care with patients. Qualitative analysis revealed three prominent thematic categories, each related to barriers PCP experienced when referring patients to palliative care: (1) PCP-level (lack of knowledge and comfort); (2) perceived patient-level (culture, family disagreement, need, home-based aspect); and (3) HBPC program-level (need to close the loop with PCP, insurance coverage, program availability, and eligibility). PCP recommendations for overcoming identified barriers will be discussed. Findings hold important implications for timely patient-referrals to palliative care by PCPs and for sustaining palliative programs that rely on these referrals.

